# Transtracheal puncture: a forgotten procedure

**DOI:** 10.1590/1414-431X20154438

**Published:** 2015-07-10

**Authors:** E.P. Almeida, A.C. Almeida, F.F. Almeida, J. Montessi, C.A. Gomes, L.E.V.V.C. Ferreira

**Affiliations:** 1 Departamento de Cirurgia, Faculdade de Medicina, Universidade Federal de Juiz de Fora, Juiz de Fora, MG, Brasil; 2 Departamento de Clínica, Faculdade de Ciências Médicas e da Saúde (FCMS-SUPREMA), Juiz de Fora, MG, Brasil; 3 Departamento de Cirurgia, Faculdade de Ciências Médicas e da Saúde (FCMS-SUPREMA), Juiz de Fora, MG, Brasil; 4 Departamento de Endoscopia, Faculdade de Medicina, Universidade Federal de Juiz de Fora, Juiz de Fora, MG, Brasil

**Keywords:** Transtracheal puncture, Aspiration, Complications, Technique, Amylase, Report values

## Abstract

Transtracheal puncture has long been known as a safe, low-cost procedure. However, with the advent of bronchoscopy, it has largely been forgotten. Two researchers have suggested the use of α-amylase activity to diagnose salivary aspiration, but the normal values of this enzyme in tracheobronchial secretions are unknown. We aimed to define the normal values of α-amylase activity in tracheobronchial secretions and verify the rate of major complications of transtracheal puncture. From October 2009 to June 2011, we prospectively evaluated 118 patients without clinical or radiological signs of salivary aspiration who underwent transtracheal puncture before bronchoscopy. The patients were sedated with a solution of lidocaine and diazepam until they reached a Ramsay sedation score of 2 or 3. We then cleaned the cervical region and anesthetized the superficial planes with lidocaine. Next, we injected 10 mL of 2% lidocaine into the tracheobronchial tree. Finally, we injected 10 mL of normal saline into the tracheobronchial tree and immediately aspirated the saline with maximum vacuum pressure to collect samples for measurement of the α-amylase level. The α-amylase level mean ± SE, median, and range were 1914 ± 240, 1056, and 24-10,000 IU/L, respectively. No major complications (peripheral desaturation, subcutaneous emphysema, cardiac arrhythmia, or hemoptysis) occurred among 118 patients who underwent this procedure. Transtracheal aspiration is a safe, low-cost procedure. We herein define for the first time the normal α-amylase levels in the tracheobronchial secretions of humans.

## Introduction

Clarke et al. (1) and Nandapalan et al. (2,3) have suggested the use of α-amylase activity as a marker of saliva aspiration. However, the normal levels of this enzyme in tracheobronchial secretions remain undefined. We prospectively evaluated 118 patients with indications for diagnostic bronchoscopy to define the levels of α-amylase activity in tracheobronchial secretions in patients without risk factors for aspiration and to study the rate of major complications of transtracheal puncture. The samples for amylase and bacteriologic assays were collected by transtracheal puncture before performing bronchoscopy. This approach was taken to avoid the introduction of nasal and oral material during the bronchoscopic examination.

Pecora (4) was the first to use transtracheal puncture to collect specimens for bacteriologic examination in patients with lung infections. His idea came from a publication by Bonica (5) in 1948 about intratracheal anesthesia. Pecora (4) performed more than 500 such procedures for diagnosis of infection without complications (4,6-8). Many other researchers have also published their experience with this method. In 1979, Pratter and Irwin (9) described the feasibility and safety of this technique. The advent of bronchoscopy in 1968 (10) caused transtracheal puncture to largely be forgotten. The objectives of the present study were to define the normal values of α-amylase activity in patients without risk of salivary aspiration using transtracheal puncture to collect specimens from the respiratory system and to determine the rate of major complications of this procedure. We evaluated patients undergoing examinations for subcutaneous emphysema, peripheral desaturation, hemoptysis, and cardiac arrhythmias.

## Material and Methods

We prospectively studied 118 patients from our thoracic service with indications for diagnostic flexible bronchoscopy for lung or pleural disease. No patients had a risk of salivary aspiration or signs of acute lung infection. The patients were sedated with a solution of 10 mg meperidine and 1 mg diazepam infused intravenously in aliquots of 2.5 mL to reach a Ramsay sedation score of 2 to 3. Antisepsis of the cervical region was performed with 70% alcohol, and the cricothyroid membrane was anesthetized with 0.5 to 1.0 mL of 2% lidocaine. A 25/7 needle was used to introduce 10 mL of lidocaine into the tracheobronchial tree to abolish the cough reflex. Finally, we introduced a catheter into a 14-G needle (BioCath, Brazil) and infused 10 mL of normal saline followed by immediate aspiration with maximum vacuum pressure to collect the tracheobronchial secretions for amylase assay. Gram staining and culture of the aspirates were performed in 20 patients. The α-amylase activity was measured using α-(2-chloro-4-nitrophenyl)-β-1,4-galactopiranomaltoside as a substrate (11). The patients were continuously monitored both clinically and using a pulse oximeter. All complications were recorded. We evaluated the development of peripheral desaturation, subcutaneous emphysema, cardiac arrhythmias, and hemoptysis. The tracheal aspirates had high viscosity, and the specimens were thus diluted; 0.1 mL of tracheal aspirate was diluted with 0.9 mL of normal saline. This project was approved by the Ethics Committee of the Universidade Federal de Juiz de Fora, MG, Brazil (protocol #0129/2009). All patients provided written informed consent.

## Results

We studied 118 patients without a risk of salivary aspiration and without clinical or radiological signs of aspiration or acute lung infection. All patients were outpatients with lung or pleural disease of unknown etiology.

The patients’ demographic data are shown in Table 1. There were no statistically significant differences in gender, race, age, or smoking history among the patients.

**Table:**
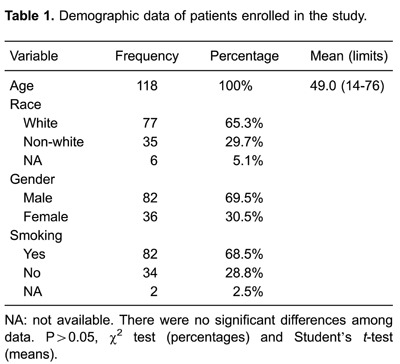

The mean ± SE and median were 1914 ± 240 IU/L and 1056 IU/L, respectively. A large variation in α-amylase activity was noted, ranging from 24 to 10,000 IU/L.

Twenty samples of transtracheal aspirates were sent for bacteriologic examination (Gram staining and culture). All aspirates had negative results.

We observed no major complications, defined as severe peripheral desaturation of hemoglobin, subcutaneous emphysema, cardiac arrhythmia, and hemoptysis. The only complication observed was minor bleeding within the tracheobronchial tree. This bleeding was asymptomatic and discovered only during bronchoscopic examination.

## Discussion

Clarke et al. (1) were the first to study α-amylase activity in tracheobronchial secretions as a possible marker of salivary aspiration. Their study showed that 6 of 21 seriously ill patients had much higher levels of α-amylase activity than did 15 patients with moderate disease (1).

In 1995, Nandapalan et al. (2,3) published two reports on α-amylase levels in tracheobronchial secretions: one involving laryngectomized patients and the other involving tracheotomized patients. The authors showed that the human lung produces amylase and stated that these lung levels should be considered as the normal α-amylase levels in humans and used to diagnose saliva aspiration.

We disagree with this information because it has been shown that physiological microaspiration occurs (12,13). Therefore, it is necessary to evaluate patients without signs of morbid aspiration to obtain normal values of α-amylase in the lungs. This is important because α-amylase could be a useful tool in the diagnosis of morbid aspiration. No studies to date have compared the α-amylase activity between aspirators and nonaspirators diagnosed by gold-standard salivary aspiration techniques (videoendoscopy or videofluoroscopy) (1,4). We believe that the first step to resolving this issue should be defining the normal levels of this enzyme, as we have done in the present study.

Since 1959, evidence has accumulated on the high value, low cost, and very low rate of complications associated with transtracheal aspiration; however, the introduction of optical fibers by Japanese researchers (10) have allowed for the visualization, biopsy, and registration of images, thus decreasing clinicians’ interest in transtracheal aspiration (4,10). However, the disadvantages of bronchoscopic techniques in patients with infection are well known (8). Bypassing the nasal and oral cavity with a very high concentration of indigenous pathogens results in difficulty differentiating colonization from infection. The availability of a low-cost, widely accessible diagnostic tool with high predictive value is important in countries such as Brazil, where bronchoscopy is only feasible in large centers, as well as for some patients who have private health insurance or require the use of public institutions. We believe that transtracheal aspiration should be an alternative option in patients with infection and a very low possibility of bronchial obstruction. The procedure should be renamed transcricoid aspiration because of the importance of avoiding puncture of the thyroid gland (4,7,9).

Like Pratter and Irwin (9), we encountered no major complications in our study. All patients had minor bleeding secondary to puncture of the cricoid membrane, but the bleeding was self-limiting and did not interfere with the patients’ blood oxygenation, which was continuously evaluated by pulse oximetry.

No positive results were obtained among the 20 transcricoid aspiration samples that we sent for Gram staining and culture. These findings are consistent with those obtained by Pecora (4,6,8) and Pecora and Kohl (7), who reported that the tracheobronchial tree is sterile in patients without infection.

## Conclusions

Transtracheal (transcricoid) aspiration is a very safe procedure. We have defined for the first time the normal levels of α-amylase activity in the tracheobronchial secretions of humans.
